# Ethnic inequities in multimorbidity among people with psychosis: a retrospective cohort study

**DOI:** 10.1017/S2045796022000385

**Published:** 2022-07-18

**Authors:** D. Fonseca de Freitas, M. Pritchard, H. Shetty, M. Khondoker, J. Nazroo, R. D. Hayes, K. Bhui

**Affiliations:** 1 Department of Psychological Medicine, Institute of Psychiatry, Psychology and Neuroscience, King's College London, London, UK; 2 Department of Psychiatry, University of Oxford, Oxford, UK; 3 Biomedical Research Centre Nucleus, South London and Maudsley NHS Foundation Trust, London, UK; 4 Norwich Medical School, University of East Anglia, Norwich, UK; 5 Sociology, School of Social Sciences, University of Manchester, Manchester, UK; 6 Nuffield Department of Primary Care Health Sciences, University of Oxford, Oxford, UK

**Keywords:** comorbidity, ethnic disparities, ethnic inequalities, ethnicity, multimorbidity, schizophrenia

## Abstract

**Aims:**

Research shows persistent ethnic inequities in mental health experiences and outcomes, with a higher incidence of illnesses among minoritised ethnic groups. People with psychosis have an increased risk of multiple long-term conditions (MLTC; multimorbidity). However, there is limited research regarding ethnic inequities in multimorbidity in people with psychosis. This study investigates ethnic inequities in physical health multimorbidity in a cohort of people with psychosis.

**Methods:**

In this retrospective cohort study, using the Clinical Records Interactive Search (CRIS) system, we identified service-users of the South London and Maudsley NHS Trust with a schizophrenia spectrum disorder, and then additional diagnoses of diabetes, hypertension, low blood pressure, overweight or obesity and rheumatoid arthritis. Logistic and multinomial logistic regressions were used to investigate ethnic inequities in odds of multimorbidity (psychosis plus one physical health condition), and multimorbidity severity (having one or two physical health conditions, or three or more conditions), compared with no additional health conditions (no multimorbidity), respectively. The regression models adjusted for age and duration of care and investigated the influence of gender and area-level deprivation.

**Results:**

On a sample of 20 800 service-users with psychosis, aged 13–65, ethnic differences were observed in the odds for multimorbidity. Controlling for sociodemographic factors and duration of care, compared to White British people, higher odds of multimorbidity were found for people of Black African [adjusted Odds Ratio = 1.41, 95% Confidence Intervals (1.23–1.56)], Black Caribbean [aOR = 1.79, 95% CI (1.58–2.03)] and Black British [aOR = 1.64, 95% CI (1.49–1.81)] ethnicity. Reduced odds were observed among people of Chinese [aOR = 0.61, 95% CI (0.43–0.88)] and Other ethnic [aOR = 0.67, 95% CI (0.59–0.76)] backgrounds. Increased odds of severe multimorbidity (three or more physical health conditions) were also observed for people of any Black background.

**Conclusions:**

Ethnic inequities are observed for multimorbidity among people with psychosis. Further research is needed to understand the aetiology and impact of these inequities. These findings support the provision of integrated health care interventions and public health preventive policies and actions.

## Introduction

Ethnic inequities^1^ in health have been reported (Sproston and Mindell, 2006; Nazroo *et al*., 2009; El-Sayed *et al*., 2011; Watkinson *et al*., 2021). For example, previous studies suggest a higher prevalence of obesity, hypertension and cardiovascular disease among Black people in the UK (Sproston and Mindell, 2006; Nazroo *et al*., 2009; El-Sayed *et al*., 2011; Schofield *et al*., 2011) and higher risk of diabetes has been observed among people of Black Caribbean, Indian, Pakistani, Bangladeshi and Chinese background (Nazroo *et al*., 2009). Evidence suggests Black and South Asian people have an earlier onset of the majority of illness (Kuan *et al*., 2019) and the prevalence of multiple long-term conditions (MLTC, or multimorbidity) is observed to be higher in almost all minoritised ethnic groups in the UK (Mathur *et al*., 2011; Bisquera *et al*., 2021; Watkinson *et al*., 2021). Also, health-related quality of life and self-rated health, a predictor of mortality, is poorer in several minoritised ethnic groups (Evandrou *et al*., 2016; Watkinson *et al*., 2021).

Ethnic health inequities have been largely associated with differences in socioeconomic status (income, education, occupation) (Nazroo and Williams, 2006; Dubath *et al*., 2021). However, evidence suggests that ethnic inequities persist after controlling for socioeconomic factors, though such models may contain residual confounding (Nazroo, 1998; Nazroo and Williams, 2006; Evandrou *et al*., 2016; Larsen *et al*., 2017; Ashworth *et al*., 2019; Verest *et al*., 2019; Watkinson *et al*., 2021). Economic adversity is related to greater exposure to negative, stressful life events (Hatch and Dohrenwend, 2007), which in turn increase the odds of worse health and multimorbidity (Lin *et al*., 2021). Minoritised ethnic groups are also more likely to face discrimination which has been associated with worse mental and physical health conditions, including psychosis, diabetes and cardiovascular diseases (Karlsen and Nazroo, 2002; Harris *et al*., 2006; Paradies *et al*., 2015; Freitas *et al*., 2018; Pearce *et al*., 2019; Oh *et al*., 2021*a*). Negative, stressful events contribute to the dysregulation of physiological functions, affecting the stress response, the immune system and leading to bodily inflammation, all of which are related to ill health (McEwen, 1998; Beckie, 2012; Goosby *et al*., 2017; O'Connor *et al*., 2021). The disruption of bodily functions in response to stress was termed as allostatic load by McEwen (1998), and it represents the wear and tear of body. Adverse life events are also related to poorer health habits (e.g. poor sleep, substance use, lack of exercise) which can lead to the onset of health conditions (Halonen *et al*., 2014; Williams *et al*., 2019). Social disadvantage, as a whole, has also been identified as a key factor in the onset of psychosis and has been suggested to be a major explanatory factor for the higher prevalence of psychosis amongst ethnic minority groups and migrants in industrialised countries (Morgan *et al*., 2019; Jongsma *et al*., 2021a, 2021b; Bhui *et al*., 2021).

People with psychosis and other severe mental illness (SMI; such as bipolar) have an elevated risk of multiple long-term conditions (or multimorbidity), with higher incidence of diabetes, hypertension, asthma, chronic obstructive pulmonary disease and chronic kidney disease (Woodhead *et al*., 2014; Stubbs *et al*., 2016; Rodrigues *et al*., 2021). However, there is limited evidence on ethnic inequities in multimorbidity among people with psychosis. A recent study reported that people from a minoritised ethnic background show an increased risk of diabetes in the first year of psychotic illness, while no major changes were observed in White people (Gaughran *et al*., 2019). No ethnicity-related difference in overall weight (body mass index), or changes in it, were observed. However, increases in central obesity (mean waist circumference) were observed among minoritised ethnic women and White men, but no major changes were observed in minoritised ethnic men or White women (Gaughran *et al*., 2019). One meta-analysis of the side-effects of antipsychotics in metabolic function reported an association between a higher proportion of non-White ethnicity and greater increases in total cholesterol; however, no significant associations between ethnicity and change in weight, body max index (BMI), LDL cholesterol, HDL cholesterol, triglycerides and glucose were observed (Pillinger *et al*., 2020).

It is important to understand if people in minoritised ethnic groups, who show a higher risk for psychosis compared to their White British counterparts, also show an excessive risk for multimorbidity when living with psychosis. This raises questions about risks for multi-disease onset, and it has implications for preventive care. As mentioned above, studies show that exposure to chronic stress leads to increased allostatic load, which contributes to the onset of physical and mental health conditions (Beckie, 2012; O'Connor *et al*., 2021). Syndemic theory also suggests that co-occurrence of health conditions is fostered by conditions of poverty and social disadvantage and will lead to worse health outcomes (Singer *et al.*, 2017). Thus, people living with mental and physical multimorbidity in conditions of social marginalisation may have a greater likelihood of intensive use of health care resources, worse quality of life and, potentially, reduced life expectancy (Das-Munshi *et al*., 2017, 2021; Kugathasan *et al*., 2020; Watkinson *et al*., 2021). To develop targeted preventive health interventions, it is important to identify if minoritised ethnic groups living with psychosis show worse physical health than their White British counterparts.

This study aims to investigate ethnic inequities in multimorbidity, and multimorbidity severity, among a cohort of people with psychosis. Acknowledging differences in multimorbidity related to gender (Larsen *et al*., 2017; Gaughran *et al*., 2019; Head *et al*., 2021; Watkinson *et al*., 2021) and socioeconomic deprivation (Marmot *et al*., 2020; Dubath *et al*., 2021; Head *et al*., 2021), we investigate interaction effects. We hypothesise that people of a minoritised ethnic background present higher multimorbidity than White British people and that this is amplified for minoritised ethnic women and minoritised ethnic people living in more deprived areas.

## Method

### Setting

This retrospective cohort study used data from the electronic health records (EHRs) of the South London and Maudsley (SLaM) National Health Service (NHS) Foundation Trust. SLaM's catchment area comprises four ethnically diverse London boroughs (Southwark, Lewisham, Lambeth and Croydon) covering 1.3 million people. Access to clinical records was obtained via the Clinical Record Interactive Search (CRIS) system (Stewart *et al*., 2009; Perera *et al*., 2016). CRIS was established under robust data protection and governance framework and received approval from the Oxford C Research Ethics Committee (18/SC/0372) to be used as a de-identified dataset for secondary data analysis (Stewart *et al*., 2009; Perera *et al*., 2016). Projects using CRIS are submitted for approval to a service-user led oversight committee; this project's reference is 20-075.

At the time of writing, CRIS enabled access to the de-identified information, in the free-text and structured fields, of the clinical records of over 400 000 service-users. One way to efficiently access and code information on the free-text fields is by using Natural Language Processing (NLP) algorithms, which eliminate the need for researchers to read and code large volumes of text (Perera *et al*., 2016; Jackson *et al*., 2017; CRIS NLP Service, 2021). These algorithms analyse text and identify the condition of interest, distinguishing true events from false positives (e.g. notes about a condition without the service-user having that condition) (Perera *et al*., 2016; Jackson *et al*., 2017). More information on the NLP algorithms used with CRIS can be found on CRIS NLP Service webpage (CRIS NLP Service, 2021). The study is reported according the RECORD statement (Benchimol *et al*., 2015).

### Participants

SLaM service-users who meet the following inclusion criteria: (i) had a schizophrenia spectrum disorder [WHO ICD-10 codes F20–F29, on structured and free-text fields of the electronic records (CRIS NLP Service, 2021)], diagnosed between 1 January 2007 and 31 December 2020, (ii) were aged between 13 and 65 years at the time of that diagnosis, (iii) had at least one clinical event between 1 January 2007 and 31 December 2020 and (iv) had information regarding ethnic identity.

### Variables

#### Multimorbidity and multimorbidity severity

Based on the current information available on CRIS we were able to identify the prevalence of six LTCs: asthma, bronchitis, diabetes, hypertension, low blood pressure, overweight or obesity and rheumatoid arthritis. Multimorbidity is commonly defined as the coexistence of two or more long-term health conditions [NICE (National Institute for Health and Care Excellence), 2016; Johnston *et al*., 2019]; so among the cohort of people with a schizophrenia spectrum disorder, multimorbidity was recognised when there was evidence of at least one physical health condition. To assess severity of multimorbidity, we further analysed the odds for the presence of one or two physical health conditions, and the presence of three or more physical health conditions.

The information regarding physical health conditions relied on the use of NLP algorithms (CRIS NLP Service, 2021) and this information was collected during the study's observation period, from 01/01/2007 to 31/12/2020. All algorithms used in this study showed good performance, with precision over 90% and recall over 76% (except for bronchitis, where recall was observed to be 48%) (CRIS NLP Service, 2021). The NLP algorithm identifying asthma analyses mentions of asthma in text. Similarly, bronchitis identification is based on statements of chronic obstructive pulmonary disease, chronic bronchitis, centrilobular emphysema or other terms related to bronchitis. Information on diabetes was derived from a combination of four sources of information: physical health forms, results from blood analysis where the haemoglobin A1c (HbA_1c_) test (which measures average blood sugar levels) indicated diabetes, and NPL algorithms to identify medications for diabetes or in-text evidence of diabetes. Hypertension was identified via the use of two NLP algorithms: one that focuses on mentions of hypertension problems in the text, and another that analyses information regarding blood pressure; high blood pressure was identified when the systolic pressure was above 140 mmHg, or the diastolic pressure was above 90 mmHg. Low blood pressure was determined when systolic pressure was below 90 mmHg, or the diastolic pressure was below 60 mmHg. To identify overweight or obesity, at any point during the observation period, we used an NLP algorithm that identified Body Mass Index (BMI) values; the information was coded as evidence of overweight or obesity when the BMI was between 25 and 50. Data regarding rheumatoid arthritis was derived from a NLP algorithm identifying mentions of rheumatoid arthritis in text (CRIS NLP Service, 2021).

#### Exposure and control variables

Ethnicity was derived from 16 NHS categories. White British ethnicity was used as a reference category in the analyses. We used most of the original NHS categories, but due to small samples, we grouped together the mixed ethnicity categories (comprising White and Black African, White Black and Caribbean and White and Asian), and grouped together the other ethnic categories (Other Ethnicity, Gypsy/Irish Traveller group and Arab). Other sociodemographic data comprised gender, age at diagnosis and area-level deprivation. Area-level deprivation was based on the English Indices of Deprivation 2015 (Department for Communities and Local Government, 2015); we retrieved the decile of the index of multiple deprivations attributed to the lower-layer super output area of the service-users address at the diagnosis date. Based on these deciles and considering the high deprivation level in the SLaM catchment area, four groups were derived: most deprived (comprising people living in deciles 1 and 2, corresponding the 20% most deprived areas in England), second-most deprived (comprising people living in decile 3 and 4), middle deprivation (comprising people living in decile 5 and 6), least deprived (comprising deciles 7 to 10, corresponding to the 40% least deprived areas).

To control for the surveillance bias associated with the availability of information, we included a measure that estimated everyone's observation period, commencing with the date of the first diagnosis of SSD after 2007 and finishing at the end of the study's observation period (31/12/2020), or date of death.

### Statistical analysis

Logistic regression analyses were conducted to investigate the association between ethnicity and multimorbidity (one or more physical health conditions) while controlling for other sociodemographic information and the observation period's length. Multinomial regression analyses were used to investigate association between ethnicity and morbidity severity, considering no multimorbidity, 1 or 2 physical health conditions and 3 or more physical health conditions. We tested interactions between ethnicity and gender and ethnicity and area-level deprivation in the predictive model for multimorbidity by using the likelihood ratio test comparing the models with and without the interaction terms, with adjustments for age, area-level deprivation and gender, respectively. Statistical analyses were conducted using STATA 15 (StataCorp, 2017).

## Results

### Participants

The sample comprises 20 800 service-users who met the inclusion criteria; 10% of those meeting the inclusion criteria were excluded because of missing or uncertain ethnicity data. Analyses revealed there were no gender differences between those excluded from the cohort due to missing data on ethnicity and the remaining cohort (47% *v*. 45% women). The median age at diagnosis for people without ethnicity data was one year earlier (35.9 *v*. 36.9 years), they were also a little more likely to live in less deprived areas (e.g. proportion living in the least deprived deciles, 7.8% *v*. 11.8%), and the length of observation period in SLaM was shorter for those with missing data on ethnicity (2.1 *v*. 8.5 years). The largest ethnic group in the cohort was people of White British ethnicity (35%), followed by Black British or other Black background (15%), Black African (14%), Black Caribbean (9%) and other White ethnicity (9%) (Table 1). The majority of the cohort comprised men (61%), and the median age at diagnosis was 37 years. Most people lived in highly deprived neighbourhoods (38% living in the 20% most deprived areas in England). Further information on the sample description is presented in Table 1.

**Table 1.** Characteristics of the study's cohort

	*N* (% on the cohort)	No multimorbidity (% on the group)	1 or more physical health conditions (% on the group)	1 or 2 physical health conditions (% on the group)	3 or more physical health conditions (% on the group)
Total	20 800 (100)	7215 (34.7)	13 585 (65.3)	8459 (40.7)	5126 (24.6)
Ethnicity					
White British	7270 (35.0)	2767 (38.1)	4503 (61.9)	2925 (40.2)	1578 (21.7)
Black British/Other Black background	3141 (15.1)	851 (27.1)	2290 (72.9)	1240 (39.5)	1050 (33.4)
Black African	2853 (13.7)	838 (29.4)	2015 (70.6)	1224 (42.9)	791 (27.7)
Black Caribbean	1825 (8.8)	423 (23.2)	1402 (76.8)	764 (41.9)	638 (35.0)
Other White background	1789 (8.6)	748 (41.1)	1065 (58.9)	766 (42.3)	299 (16.6)
Asian British/Other Asian background	848 (4.1)	314 (37.0)	534 (63.0)	333 (39.3)	201 (23.7)
Mixed ethnic background	689 (3.3)	244 (35.4)	445 (64.6)	274 (39.8)	171 (24.8)
Irish	333 (1.6)	127 (38.1)	206 (61.9)	128 (38.4)	78 (23.4)
Indian	327 (1.6)	103 (31.5)	224 (68.5)	142 (43.4)	82 (25.1)
Pakistani	207 (1.0)	80 (38.7)	127 (61.4)	90 (43.5)	37 (17.9)
Chinese	134 (0.6)	65 (48.5)	69 (51.5)	48 (35.8)	21 (15.7)
Bangladeshi	125 (0.6)	50 (40.0)	75 (60.0)	51 (40.1)	24 (19.2)
Other ethnic background	1259 (6.1)	605 (49.1)	630 (50.9)	474 (38.4)	156 (12.6)
Gender					
Men	12 593 (60.5)	4352 (34.6)	8241 (65.4)	5023 (39.9)	3218 (25.6)
Women	8207 (39.5)	2863 (34.9)	5344 (65.1)	3436 (41.9)	1908 (23.3)
Age: *Mdn (IQR)*	36.9 (27.0–47.1)	35.6 (26.9–45.7)	37.6 (27.0–47.8)	37.8 (27.3–48.1)	37.1 (27.3–48.1)
13–17 years	1018 (4.9)	399 (39.2)	619 (60.8)	412 (40.47)	207 (20.3)
18–34 years	8367 (40.2)	3076 (36.8)	5291 (63.2)	3232 (38.6)	2059 (24.6)
35–49 years	7404 (35.6)	2256 (34.1)	4878 (65.9)	3018 (40.8)	1860 (25.1)
50–65 years	4011 (19.3)	1214 (30.27)	27 978 (69.7)	1797 (44.8)	1000 (24.9)
Area-level deprivation ^a^ (7.4% missing data)					
Least deprived (IMD deciles 7–10)	1497 (7.8)	634 (42.4)	863 (57.7)	606 (40.5)	257 (17.2)
Middle deprivation (IMD deciles 5–6)	3141 (16.3)	1093 (34.8)	2048 (65.2)	1288 (41.0)	760 (24.2)
Second most deprived (IMD deciles 3–4)	7300 (37.9)	2470 (33.8)	4830 (66.2)	2939 (40.3)	1891 (25.9)
Most deprived (IMD deciles 1–2)	7328 (38.0)	2391 (32.6)	4937 (67.4)	3019 (41.2)	1918 (26.2)
Observation period (years): *M* (*SD*)	8.11 (4.40)	7.31 (4.35)	8.55 (4.37)	7.83 (4.43)	9.74 (4.00)
*Physical health conditions*		*% on multimorbidity group*			
Asthma	3119 (15.0)	0	3119 (23)	1126 (13.3)	1993 (38.9)
Bronchitis	552 (2.7)	0	552 (4.1)	115 (1.4)	437 (8.5)
Diabetes	6687 (32.2)	0	6687 (49.2)	2751 (32.5)	3936 (76.8)
Hypertension/high blood pressure	9481 (45.6)	0	9481 (59.8)	4712 (55.7)	4769 (93.0)
Low blood pressure	5112 (24.6)	0	5112 (37.6)	1616 (19.1)	3496 (68.2)
Overweight or obesity	5438 (26.1)	0	5438 (40.0)	1817 (21.5)	3621 (70.6)
Rheumatoid arthritis	172 (0.8)	0	172 (1.3)	57 (0.7)	115 (2.2)

*Notes*: ^a^Proportions for area-level deprivation were calculated from available data.

The most prevalent conditions were hypertension (46%), followed by diabetes (32%), overweight or obesity (26%), low blood pressure (25%) and asthma (15%); bronchitis (3%) and rheumatoid arthritis (1%) were not common (Table 1). Information on the sociodemographic and clinical factors stratified by ethnicity are presented in Table 2.

**Table 2.** Ethnic differences in the sociodemographic factors and health conditions

% within ethnicity	White British	Black British	Black African	Black Caribbean	Other White	Asian British	Mixed ethnicity	Irish	Indian	Pakistani	Chinese	Bangladeshi	Other ethnicity	*p*-value
Women (Gender)	38.1	38.7	42.7	40.5	38.6	39.4	43.8	29.1	39.5	43.5	60.5	40.8	38.9	<0.001
Age: *Mdn (IQR)*	39.4 (28.6–50.5)	32.7 (24.0–42.9)	34.9 (26.3–43.9)	42.8 (31.9–50.5)	35.3 (28.1–44.3)	36.1 (26.7–45.3)	29.8 (22.0–40.6)	43.0 (32.5–52.9)	41.3 (32.1–51.3)	32.6 (24.9–43.1)	32.1 (26.3–41.7)	31.0 (25.3–39.2)	34.9 (27.0–45.5)	
13–17 years	5.0	6.7	3.8	3.3	2.6	5.1	12.8	3.9	3.4	7.25	4.5	4.8	3.4	<0.001
18–34 years	34.0	48.4	46.5	27.0	46.5	42.1	48.6	28.5	29.7	48.8	55.2	60.0	46.7	
35–49 years	34.7	35.6	37.1	43.0	35.1	35.7	28.6	37.5	37.9	28.0	28.4	28.8	33.0	
50–65 years	26.3	9.3	12.7	26.7	15.8	17.1	10.0	30.0	29.1	15.9	11.9	6.4	16.8	
Area-level deprivation														<0.001
Least deprived (7–10 deciles)	12.9	3.8	2.6	3.6	8.7	8.2	8.8	6.5	6.4	4.6	9.2	7.8	5.3	
Middle deprivation (5–6 deciles)	18.7	15.0	11.0	15.5	17.9	14.8	15.5	15.7	22.0	25.8	17.7	14.8	15.2	
Second most deprived (3–4 deciles)	34.7	40.7	38.8	38.8	36.9	41.3	40.3	40.82	45.5	43.9	34.5	30.4	40.7	
Most deprived (1–2 deciles)	33.7	40.5	47.6	42.1	36.5	35.7	35.4	37.1	26.1	25.8	38.7	47.0	38.8	
Observation period (years): *M* (*SD*)	7.78 (4.42)	8.44 (4.44)	8.56 (4.33)	9.70 (4.29)	7.51 (4.32)	8.26 (4.28)	7.82 (4.59)	8.37 (4.28)	8.46 (4.40)	7.32 (4.49)	8.70 (4.04)	8.04 (4.12)	6.84 (3.82)	<0.001
*Physical health comorbidities*														
Asthma	16.2	19.9	11.2	17.9	10.3	12.0	18.6	17.4	15.3	12.6	6.7	10.4	8.1	<0.001
Bronchitis	4.0	2.0	1.5	5.9	2.0	2.0	2.0	4.2	1.8	2.4	1.5	0.8	0.6	<0.001
Diabetes	27.7	38.9	35.9	46.5	23.8	35.9	33.0	28.5	39.8	30.4	22.4	34.4	20.4	<0.001
Hypertension	41.8	53.7	53.0	60.3	36.5	40.9	44.0	41.4	49.2	35.3	35.1	34.4	30.3	<0.001
Low blood pressure	22.2	31.5	26.9	28.7	21.4	25.0	25.5	22.8	20.5	21.7	24.6	17.6	16.5	<0.001
Overweight or obesity	22.2	33.6	30.9	32.6	22.1	25.4	25.1	23.1	22.0	24.6	20.9	28.8	19.6	<0.001
Rheumatoid arthritis	1.1	0.7	0.6	1.2	0.6	0.5	1.0	0.9	0.6	1.0	0.0	00.0	0.6	0.165

### Ethnicity and odds for multimorbidity

The results from the logistic regression analyses (Table 3) showed that adjusting for age, gender, area-level deprivation and observation period, compared to White British people, Black African [aOR = 1.41, 95% CI (1.23–1.56)], Black Caribbean [aOR = 1.79, 95% CI (1.58–2.03)] and Black British people [aOR = 1.64, 95% CI (1.49–1.81)] were more likely to have multimorbidity (psychosis plus one physical health condition). Reduced odds for multimorbidity were observed among people of Chinese [aOR = 0.61, 95% CI (0.43–0.88)] and Other ethnicities [aOR = 0.66, 95% CI (0.58–0.75)]. The magnitude of these differences was even higher when comparing the odds for severe multimorbidity, having 3 or more physical health conditions (Table 3). No significant differences in odds for multimorbidity were observed between White British and people of South Asian background or Asian British, Irish, other White background and mixed race. However, in the model regarding multimorbidity severity, people of White Other ethnicity were observed to have reduced odds for 3 or more physical health conditions [aOR = 0.78, 95% CI (0.66–0.92)], compared to White British people. The effects of ethnicity on multimorbidity did not differ by gender or level of deprivation as indicated by the statistically non-significant interaction terms in the logistic models [likelihood ratio (LR) test based chi-squared = 14.45(12), *p* = 0.273 for ethnicity × gender and 44.48(36), *p* = 0.148 for ethnicity × deprivation interactions respectively].

**Table 3.** Odd ratios (OR) for having multimorbidity and multimorbidity severity compared to having no physical health conditions

	1 or more physical health conditions		1 or 2 physical health conditions	3 or more physical health conditions
	Crude OR [95% CI]^a^	Adjusted OR [95% CI]^a^	Adjusted OR [95% CI]^b^	Adjusted OR [95% CI]^b^
Ethnicity				
White British	Ref	Ref	Ref	Ref
Black British/Other Black background	**1.65** [**1.51–1.81]**	**1.64** [**1.49–1.81]**	**1.42** [**1.27–1.58]**	**2.06** [**1.83–2.31]**
Black African	**1.48 [1.35–1.62]**	**1.41 [1.23–1.56]**	**1.36 [1.22–1.52]**	**1.50 [1.33–1.70]**
Black Caribbean	**2.04 [1.81–2.29]**	**1.79 [1.58–2.03]**	**1.61 [1.40–1.84]**	**2.09 [1.81–2.42]**
Other White background	**0.88 [0.79–0.98]**	0.95 [0.84–1.07]	1.04 [0.91–1.18]	**0.78 [0.66–0.92]**
Asian British/Other Asian background	1.05 [0.90–1.21]	1.05 [0.90–1.23]	1.05 [0.88–1.24]	1.06 [0.87–1.30]
Irish	1.00 [0.79–1.25]	0.94 [0.74–1.21]	0.90 [0.69–1.19]	1.02 [0.75–1.39]
Indian	**1.34 [1.05–1.70]**	1.25 [0.97–1.60]	1.25 [0.96–1.63]	1.26 [0.92–1.71]
Pakistani	0.98 [0.73–1.30]	1.03 [0.76–1.38]	1.09 [0.80–1.50]	0.89 [0.59–1.33]
Chinese	**0.65 [0.46–0.92]**	**0.61 [0.43–0.88]**	0.70 [0.47–1.04]	**0.47 [0.27–0.81]**
Bangladeshi	0.92 [0.64–1.32]	0.96 [0.66–1.42]	1.07 [0.71–1.61]	0.78 [0.46–1.33]
Mixed ethnic background	1.12 [0.95–1.32]	1.15 [0.97–1.36]	1.11 [0.92–1.34]	1.22 [0.98–1.52]
Other ethnic background	**0.64 [0.57–0.72]**	**0.66 [0.58–0.75]**	**0.75 [0.65–0.86]**	**0.50 [0.41–0.61]**
Gender				
Men	Ref	Ref	Ref	Ref
Women	0.99 [0.92–1.04]	0.97 [0.92–1.04]	1.02 [0.95–1.09]	**0.90 [0.83–0.98]**
Age				
13–17 years	Ref	Ref	Ref	Ref
18–34 years	1.11 [0.97–1.27]	1.05 [0.91–1.21]	0.99 [0.86–1.16]	1.15 [0.96–1.39]
35–49 years	**1.24 [1.09–1.42]**	1.13 [0.99–1.31]	1.12 [0.97–1.31]	1.14 [0.95–1.38]
50–65 years	**1.49 [1.29–1.71]**	**1.53 [1.32–1.78]**	**1.49 [1.27–1.75]**	**1.60 [1.31–1.96]**
Area-level deprivation				
Least deprived (IMD deciles 7–10)	Ref	Ref	Ref	Ref
Middle deprivation (IMD deciles 5–6)	**1.38 [1.21–1.56]**	**1.26 [1.11–1.43]**	**1.17 [1.02–1.34]**	**1.50 [1.25–1.79]**
Second most deprived (IMD deciles 3–4)	**1.44 [1.28–1.61]**	**1.27 [1.13–1.43]**	**1.15 [1.02–1.31]**	**1.56 [1.32–1.84]**
Most deprived (IMD deciles 1–2)	**1.52 [1.35–1.70]**	**1.32 [1.18–1.49]**	**1.21 [1.07–1.37]**	**1.61 [1.37–1.90]**
Observation period (years)	**1.07 [1.06–1.07]**	**1.06 [1.05–1.07]**	**1.02 [1.02–1.03]**	**1.13 [1.12–1.14]**

*Notes*: a – results from logistic regression; b – results from multinomial regression. Significant results are indicated in bold.

## Discussion

We investigated ethnic inequities in risks of multimorbidity in people with psychosis, using data from the electronic health records of South London and Maudsley NHS Trust. Black African, Black Caribbean and Black British people with psychosis have around 1.5 greater odds for multimorbidity, and around twice the odds for severe multimorbidity (i.e. psychosis plus three physical health conditions). People of Chinese ethnicity and Other ethnicity had reduced odds for multimorbidity. This is the first study that investigates potential ethnic inequities in multimorbidity among a cohort of people with psychosis. These findings are partly in line with a study that observed a greater risk of the development of diabetes among minoritised ethnic people within the first year of treatment for psychosis (Gaughran *et al*., 2019). Also, the pattern of observed inequities is partially similar to a study of multimorbidity among a national sample of older adults (Watkinson *et al*., 2021). Similar findings are the greater risk observed among Black people, the reduced odds among Chinese people and the absence of differences among people with Mixed ethnicity (Watkinson *et al*., 2021). However, in our study, we did not observe increased odds for multimorbidity among people of South Asian, Asian British or other ethnicities. The age difference of the samples and the fact that we only investigated six health conditions, while the study of multimorbidity among older adults investigated 14 (Watkinson *et al*., 2021), may be the reasons for differences in findings.

Multiple studies have investigated the reasons for ethnic inequities in health. Except for a few conditions (e.g. sickle cell anaemia) that have a simple genetic basis, studies have failed to provide sound biological explanations for the ethnic inequities observed (Dressler *et al*., 2005; Adler and Rehkopf, 2008). Geographical variance in the prevalence of health conditions, as well as the significant associations between area deprivation, income, education and occupation with multimorbidity, suggest that differences in health and multimorbidity are more likely rooted in social conditions than are due to innate biological vulnerabilities (Nazroo, 1998; Evandrou *et al*., 2016; Stubbs *et al*., 2016; Larsen *et al*., 2017; Verest *et al*., 2019; Dugravot *et al*., 2020; Dubath *et al*., 2021). Studies show that exposure to stress is associated with the dysregulation of the hypothalamic-pituitary-adrenal (HPA) axis and related cortisol awakening response (CAR), with implications for metabolic and inflammatory processes, further dysregulation of the immune response and multi-illness onset (O'Connor *et al*., 2021). Furthermore, social epigenetics studies report evidence of change in DNA methylation leading to epigenetic ageing amongst people who faced childhood adversity, low socioeconomic status in childhood and adulthood, perceived discrimination and neighbourhood disadvantage (Martin *et al*., 2022).

The observed ethnic inequities could probably be driven by inequities in the social determinants of health beyond the included measure of area deprivation, including social marginalisation, racial discrimination, and, for migrants, the stresses related to migration and acculturation (Hatch and Dohrenwend, 2007; Allen *et al*., 2014; Cabinet Office, 2018; Jongsma *et al*., 2021b; Marmot *et al*., 2020; Nazroo *et al*., 2020; Bhui *et al*., 2021). For instance, experiences of racism and racial discrimination have been related to worse mental and physical health, including the onset of psychosis, diabetes, respiratory illness and hypertension (Karlsen and Nazroo, 2002; Karlsen *et al*., 2005; Paradies *et al*., 2015; Pearce *et al*., 2019; Williams *et al*., 2019; Jongsma *et al*., 2021a). Health-related behaviours could mediate the relationships between social adversity and the onset of multimorbidity (Goosby *et al*., 2017; Williams *et al*., 2019; Bhui *et al*., 2021). The findings may also reflect the shared aetiology of mental and physical health conditions. One study has reported an association between the number of psychotic symptoms and number of physical health conditions (Moreno *et al*., 2013). The role of social stress, allostatic load, bodily inflammation and coagulation have been related to the onset of disorders in physical health, as well as psychosis; these mechanisms may explain multimorbidity with psychosis (Beckie, 2012; Goosby *et al*., 2017; Howes and McCutcheon, 2017; Lund *et al*., 2018; Heurich *et al*., 2022).

This study has implications for integrated care and shows the need for developing targeted interventions to reduced ethnic inequities. Literature suggests there is room for improving the health care provided. Ethnic minority people may not be disadvantaged in terms of contacts with the medical health system; a national representative study shows that after adjusting for self-assessed level of health and number of long-term conditions, people of Caribbean, Indian, Pakistani and Bangladeshi backgrounds were more likely to have had an appointment in the two weeks prior the survey (Nazroo *et al*., 2009). However, there is evidence that Asian older adults are more dissatisfied with the care provided by GPs, as compared to White British, and older adults of most ethnic groups report insufficient support from local services to manage their health conditions and have low confidence in that management (Watkinson *et al*., 2021).

There are specific barriers to managing physical health among people with psychosis, which range from increased individual risk factors for poorer health (e.g. higher rates of substance use, smoking, lack of exercise and poorer diet), to service-related factors (e.g. lack of clarity in who is responsible for managing health problems among people with psychosis) (De Hert *et al*., 2011; Moore *et al*., 2015; Bellass *et al*., 2021). These barriers can be aggravated for ethnic minority people due to discrimination, difficulties in communication or other cultural barriers. There is evidence that Black people with an SMI are less likely than White counterparts to have psychiatric diagnostic information recorded in hospital records after an emergency hospital admission (Mansour *et al*., 2020). This under-identification potentially is related to reduced opportunities for enhanced continuity of care. The emphasise on increasing comprehensive personalised care may be a useful resource to reduce the ethnic inequities in multimorbidity (as it aims to improve individual levels of self-management of chronic health conditions, as well as to promote opportunities for people to have more community support), and evidence suggests that this leads to improved health outcomes (Coulter *et al*., 2015). However, information on how people with psychosis manage their physical health and any specific initiatives to reduce ethnic inequities in multimorbidity amongst people with psychosis is lacking (Das-Munshi *et al*., 2016). This study identifies the need to promote integrated care for SMI and multimorbidity (De Hert *et al*., 2011; Moore *et al*., 2015; Bellass *et al*., 2021).

### Strengths and limitations

This study used data from one of the UK's largest secondary mental health facilities, serving an ethnically diverse community. The cohort of the study is representative of South London and Maudsley NHS Trust catchment, and the findings should be generalisable to other ethnically dense urban areas in the United Kingdom. Indeed, most ethnic minorities live in urban areas. Ethnicity was self-defined, within NHS categories; however we could not include other relevant factors that can be related to inequities in health, most importantly migration, which could be associated with potential related barriers in the use of health services and under-detection of health conditions, but also an investigation of the healthy migrant paradox (Helgesson *et al*., 2019; Oh *et al*., 2021*b*). Also, we could not adjust for individual measures socioeconomic status (e.g. income, level of education), experiences of racism and discrimination or other factors of social adversity which have been associated with health inequities (Karlsen and Nazroo, 2002; Beckie, 2012; Goosby *et al*., 2017). There are limitations in using Natural Language Processing (NLP) algorithms to assess the prevalence of physical health conditions, however the use of NLP enabled us to investigate ethnic inequities in a larger sample of people than what would be possible if restricting to people whose data could be linked to other administrative datasets that contain data on physical health, such as the Lambeth DataNet (Woodhead *et al*., 2014). There is the risk of underestimation of the prevalence of some physical health conditions, as they would be managed in primary care and not recorded in SLaM (Gaughran *et al*., 2019). We did not account for potential ethnic differences in psychotic illness severity and healthcare service use (Chui *et al*., 2021; Morris *et al*., 2020), which may affect the availability of information. Moreover, only the conditions relevant to the aetiology and management of psychosis may be reported in SLaM electronic records, so we may have less information on other conditions. We did not adjust for the type of pharmacological treatment for psychosis, which could be related to the presence of the included physical conditions (Baxter *et al*., 2016; Dubath *et al*., 2021). Finally, we could not investigate the timing of the onset of the different health conditions. Future cohort studies are needed to verify and expand on our findings (e.g. Das-Munshi *et al*., 2016).

## Conclusions

This study shows that Black people with psychosis are at higher risk of multimorbidity. Although this study is not focused on aetiology, the findings are consistent with adversity-related onset of disease, where marginalised ethnic groups face higher rates of psychosis and other health conditions when living with this severe mental illness (Dubath *et al*., 2021; Jongsma *et al*., 2021a; Watkinson *et al*., 2021). The observed ethnic inequities in health show the need for integrated care and multi-disease prevention among minoritised ethnic groups.

## Data Availability

The data that support the findings of this study are available on request from the corresponding author. The data are not publicly available due to the Information Governance framework and Research Ethics Committee approval in place concerning CRIS data use.

## Footnotes

1 We use the term inequity in line with the World Health Organisation definition of health equity as ‘the absence of unfair, avoidable or remediable differences among groups of people, whether those groups are defined socially, economically, demographically, or geographically or by other dimensions of inequality (e.g. sex, gender, ethnicity, disability, or sexual orientation).’ (World Health Organization, 2021).
